# Supplementary material for: Towards unified AI-driven fracture mechanics: the extended deep energy method (XDEM)
Source: Nat Commun. 2026 Aug 15;17:8492. doi: 10.1038/s41467-026-76748-1 (PMC13484471; doi:10.1038/s41467-026-76748-1)
Supplement: Supplementary file 3 [file 41467_2026_76748_MOESM3_ESM.zip › pic/Results/Stress_intensity_factor/Mixed/Figure_2.pdf]

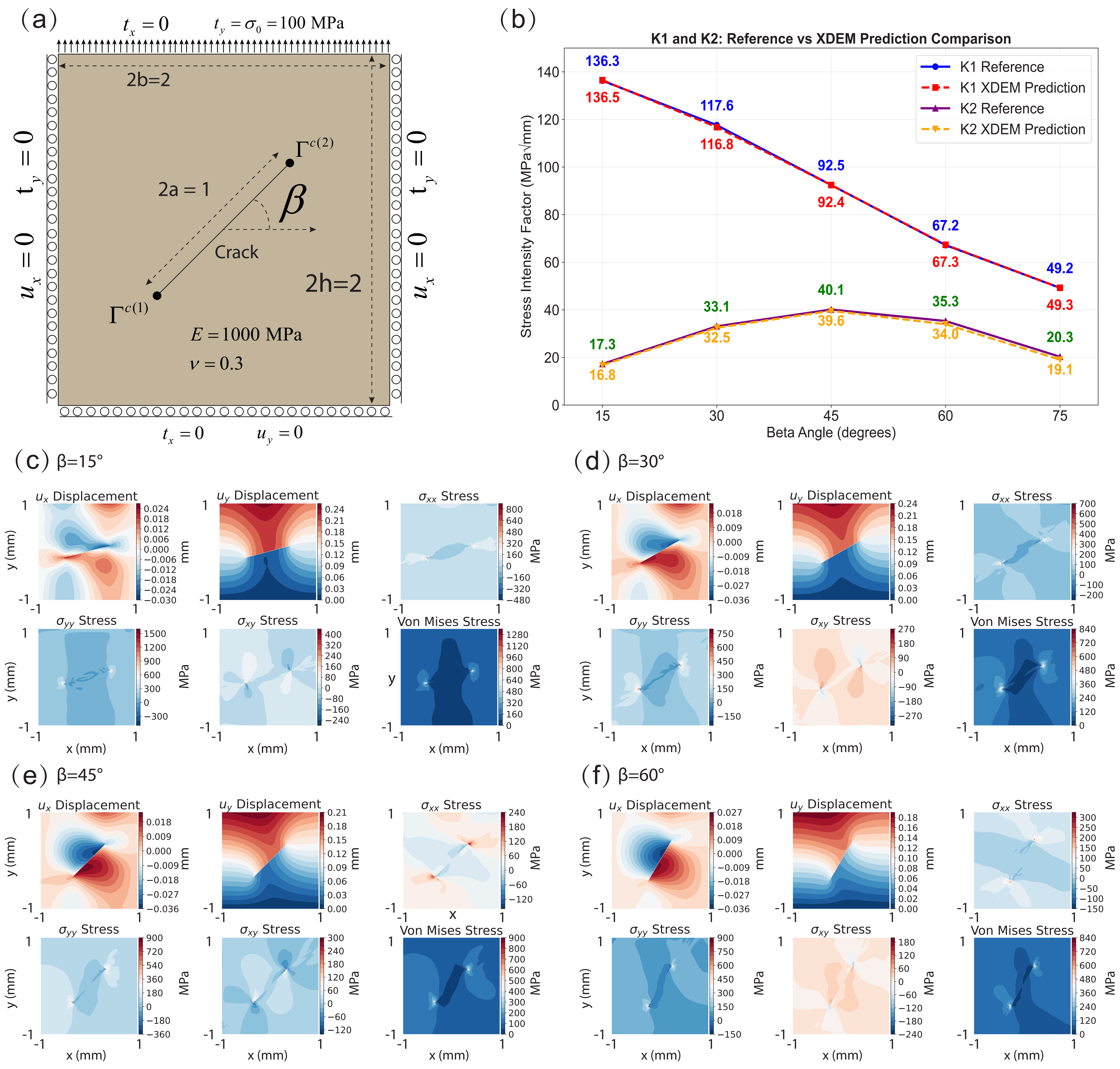

(a)
$t_x = 0$ $t_y = \sigma_0 = 100$ MPa
2b=2
$\Gamma^{c(2)}$
2a = 1
β
Crack
2h=2
$\Gamma^{c(1)}$
$E = 1000$ MPa
$\nu = 0.3$
$u_x = 0$ $t_y = 0$
$t_x = 0$ $u_y = 0$
(b)
K1 and K2: Reference vs XDEM Prediction Comparison
K1 Reference
K1 XDEM Prediction
K2 Reference
K2 XDEM Prediction
Stress Intensity Factor (MPa√mm)
Beta Angle (degrees)
136.3
136.5
117.6
116.8
92.5
92.4
67.2
67.3
49.2
49.3
17.3
16.8
33.1
32.5
40.1
39.6
35.3
34.0
20.3
19.1
(c) β=15°
(d) β=30°
(e) β=45°
(f) β=60°
u_x Displacement
u_y Displacement
σ_{xx} Stress
σ_{yy} Stress
σ_{xy} Stress
Von Mises Stress
x (mm)
y (mm)
mm
MPa
